# Supplementary material for: Uncovering tissue-specific endophytic microbiota composition and activity in Rhizophora mangle L.: a metagenomic and metatranscriptomic approach
Source: PeerJ. 2025 Aug 28;13:e19728. doi: 10.7717/peerj.19728 (PMC12399087; doi:10.7717/peerj.19728)
Supplement: Supplemental Information 1 [file peerj-13-19728-s001.docx]

| **Meteorological parameters** | **Results** | **Unit** |
| --- | --- | --- |
| **Temperature** | 30.2 | °C |
| **Heat index** | 39 | °C |
| **Cold index** | 30 | °C |
| **Dew point** | 26.3 | °C |
| **Wet bulb** | 27.3 | °C |
| **Humidity** | 78 | % |
| **Pressure** | 14.65 | PSI |
| **Barometer** | 13.24 | PSI |
| **Height** | -9.5 | masl |
