# Supplementary material for: Uncovering tissue-specific endophytic microbiota composition and activity in Rhizophora mangle L.: a metagenomic and metatranscriptomic approach
Source: PeerJ. 2025 Aug 28;13:e19728. doi: 10.7717/peerj.19728 (PMC12399087; doi:10.7717/peerj.19728)
Supplement: Supplemental Information 2 [file peerj-13-19728-s002.docx]

| **Soil physicochemical parameters** | | | **Water physicochemical parameters** | | |
| --- | --- | --- | --- | --- | --- |
| **Parameter** | **Result** | **Unit** | **Parameter** | **Result** | **Unit** |
| **pH** | 5.91 | pH | **pH** | 7.42 | pH |
| **Electrical conductivity** | 30.2 | dS/m | **Redox potential** | 119.4 | mV |
| **Oxidizable organic carbon** | 1.78 | % | **Oxygen saturation** | 81.3 | % |
| **Organic matter** | 3.07 | % | **Dissolved oxygen** | 5.14 | ppm |
| **Total nitrogen** | 0.148 | % | **Conductivity** | 41.4 | mS/cm |
| **Exchangeable potassium** | 642 | mg/kg | **Conductivity** | 71.7 | mS/cmA |
| **Exchangeable calcium** | 792 | mg/kg | **Resistivity** | 0.8 | Ω-cm |
| **Interchangeable magnesium** | 994 | mg/kg | **Total dissolved solids** | 24.14 | ppm |
| **Exchangeable sodium** | 4480 | mg/kg | **Salinity** | 18.5 | σT |
| **Phospohorus** | 20.5 | mg/kg | **Turbidity** | 38.4 | cm |
| **Sulfur** | 489 | mg/kg | **Temperature** | 30.4 | °C |
