# Supplementary material for: Uncovering tissue-specific endophytic microbiota composition and activity in Rhizophora mangle L.: a metagenomic and metatranscriptomic approach
Source: PeerJ. 2025 Aug 28;13:e19728. doi: 10.7717/peerj.19728 (PMC12399087; doi:10.7717/peerj.19728)
Supplement: Supplemental Information 3 [file peerj-13-19728-s003.docx]

| **Sample** | **Number of DNA reads (M)** | | **Number or RNA reads (M)** | |
| --- | --- | --- | --- | --- |
|  | **Raw reads** | **Processed reads** | **Raw reads** | **Processed reads** |
| **RmL1** | 82663806 | 1013219 | 97557880 | 1280356 |
| **RmL2** | 81367835 | 1108079 | 73187512 | 697818 |
| **RmL3** | 81050361 | 1358879 | 76745492 | 655212 |
| **RmR1** | 83438373 | 18012580 | 78466460 | 1875656 |
| **RmR2** | 69261870 | 9386408 | 79177582 | 957354 |
| **RmR3** | 83575760 | 23512378 | * | * |
| **Total** | 481358005 | 54391543 | 405134926 | 5466396 |

*RNA RmR3 replication is not included, see methodology for details.
