# Supplementary figures and images for: Uncovering tissue-specific endophytic microbiota composition and activity in Rhizophora mangle L.: a metagenomic and metatranscriptomic approach
Source: PeerJ. 2025 Aug 28;13:e19728. doi: 10.7717/peerj.19728 (PMC12399087; doi:10.7717/peerj.19728)

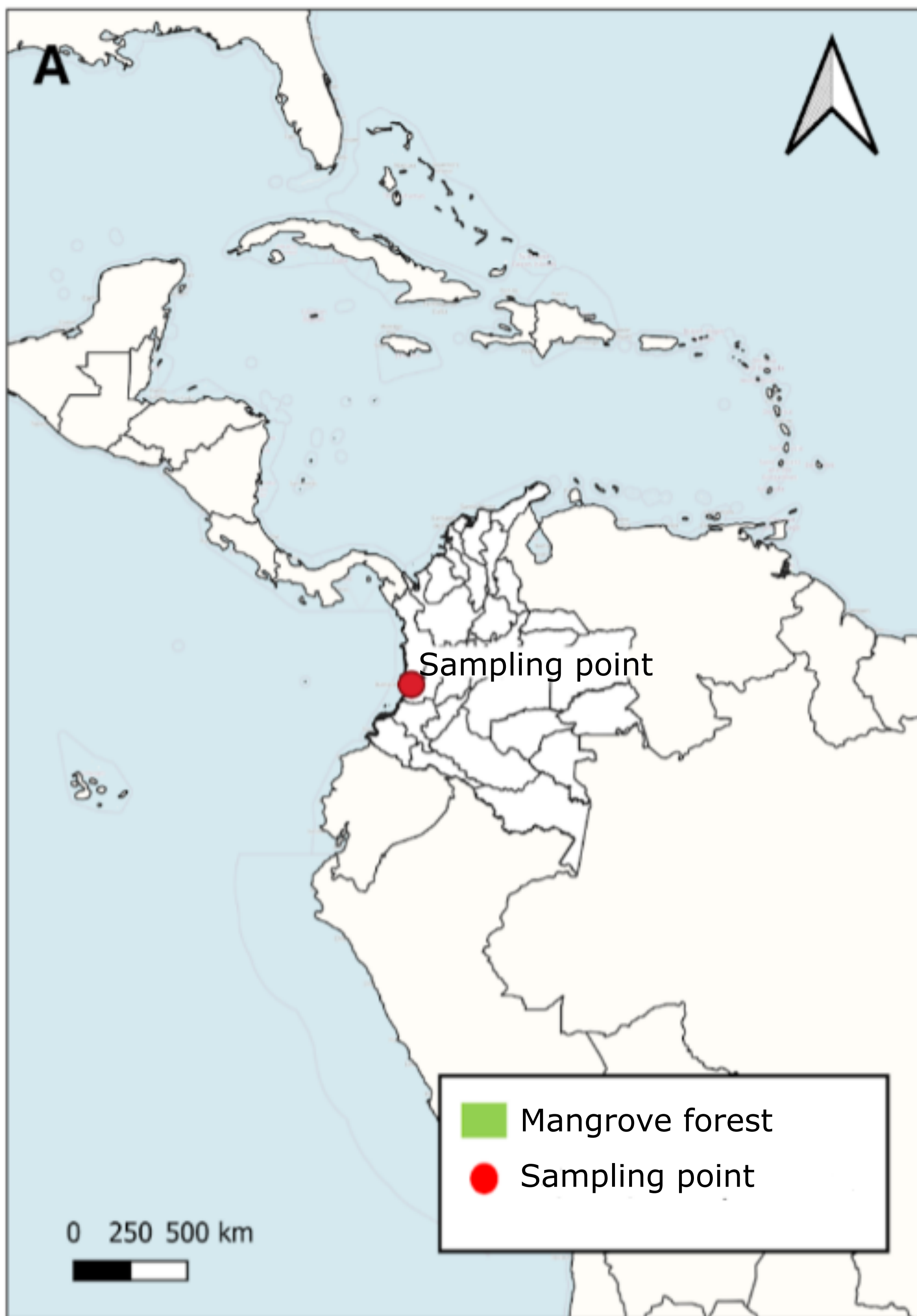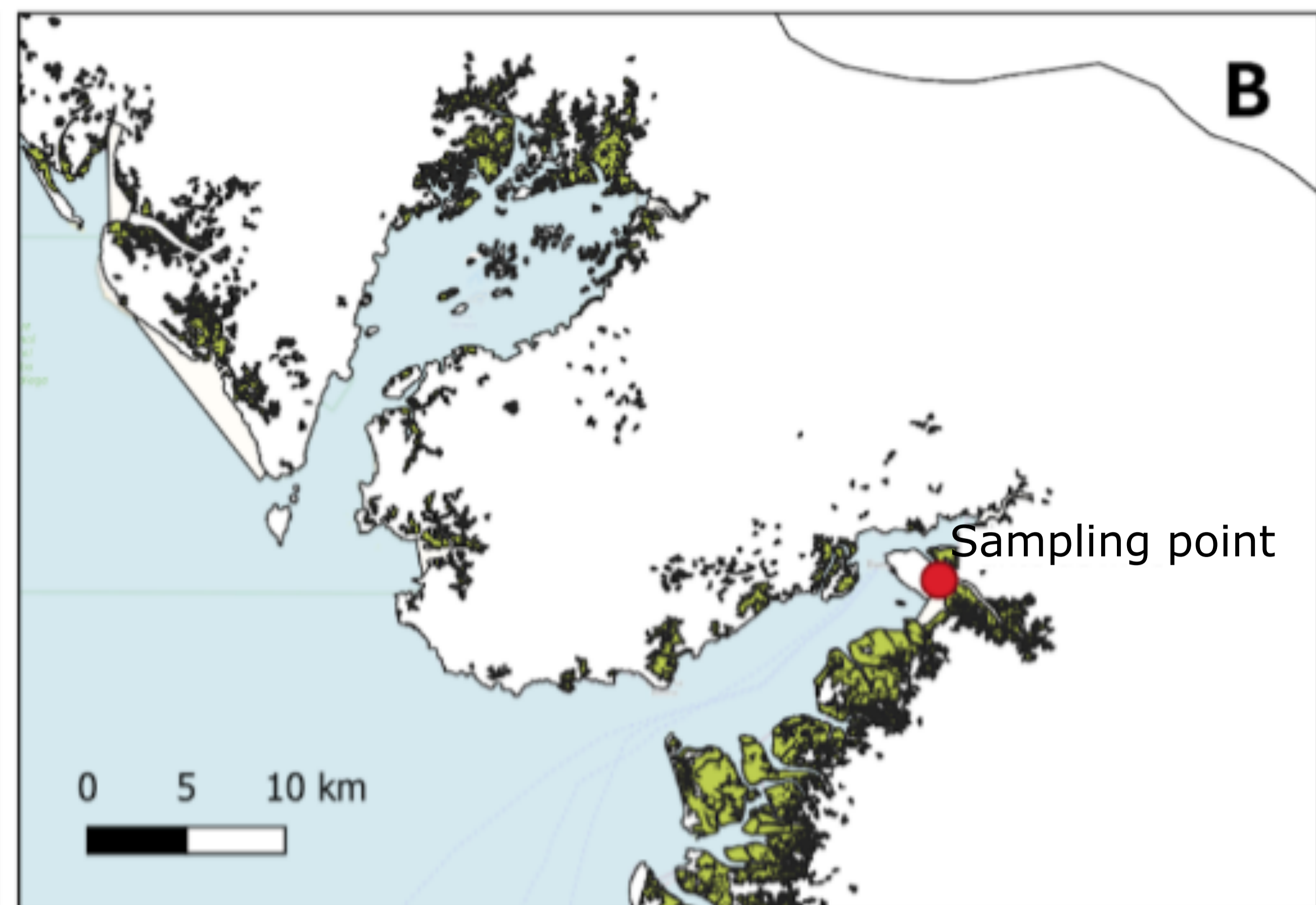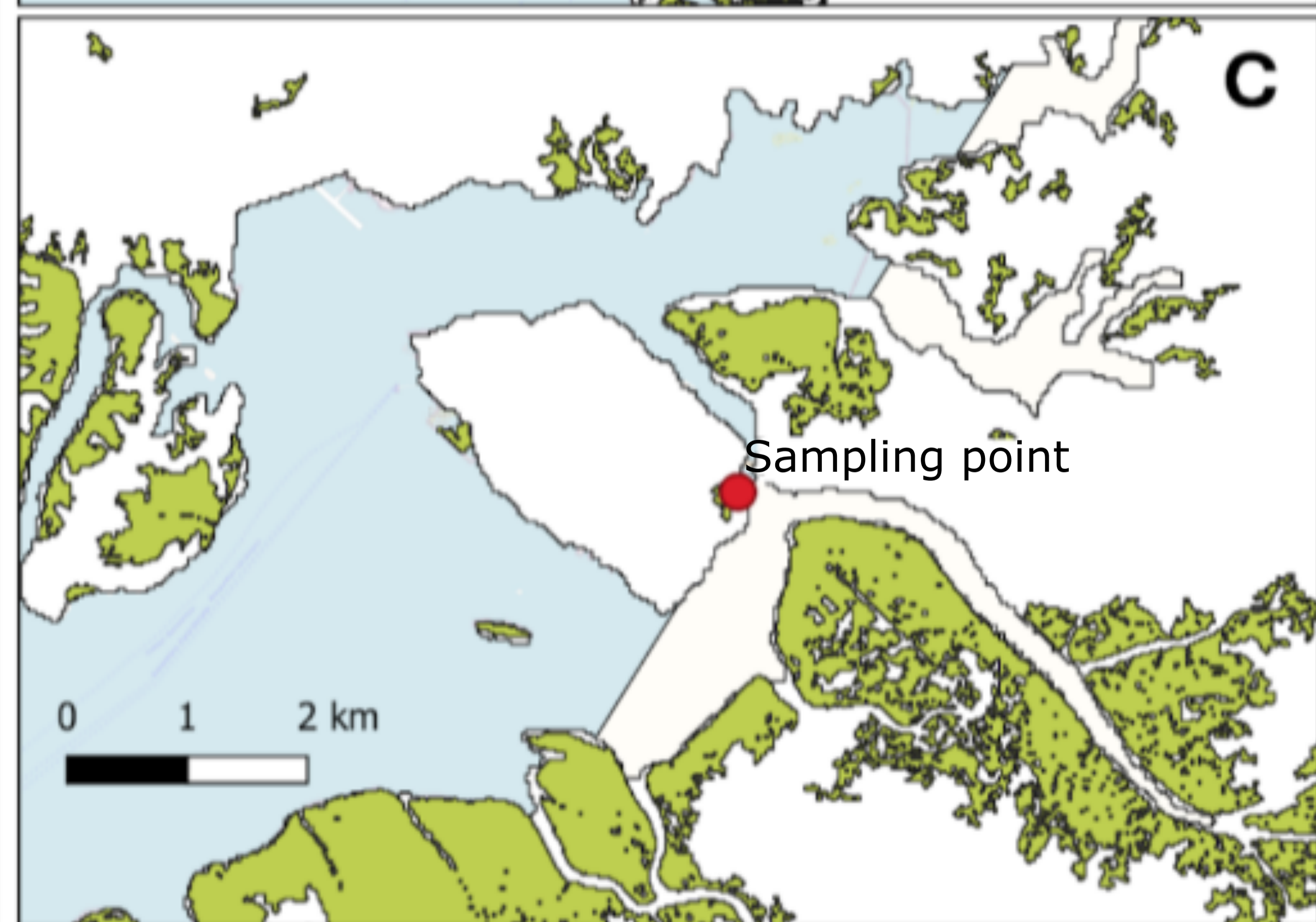

Supplement: Supplemental Information 5 — A). Buenaventura Bay in southwestern Colombia. B) Dagua River estuary into the Pacific Ocean. C) Sampling area inside the mangrove forest. [file peerj-13-19728-s005.pdf]

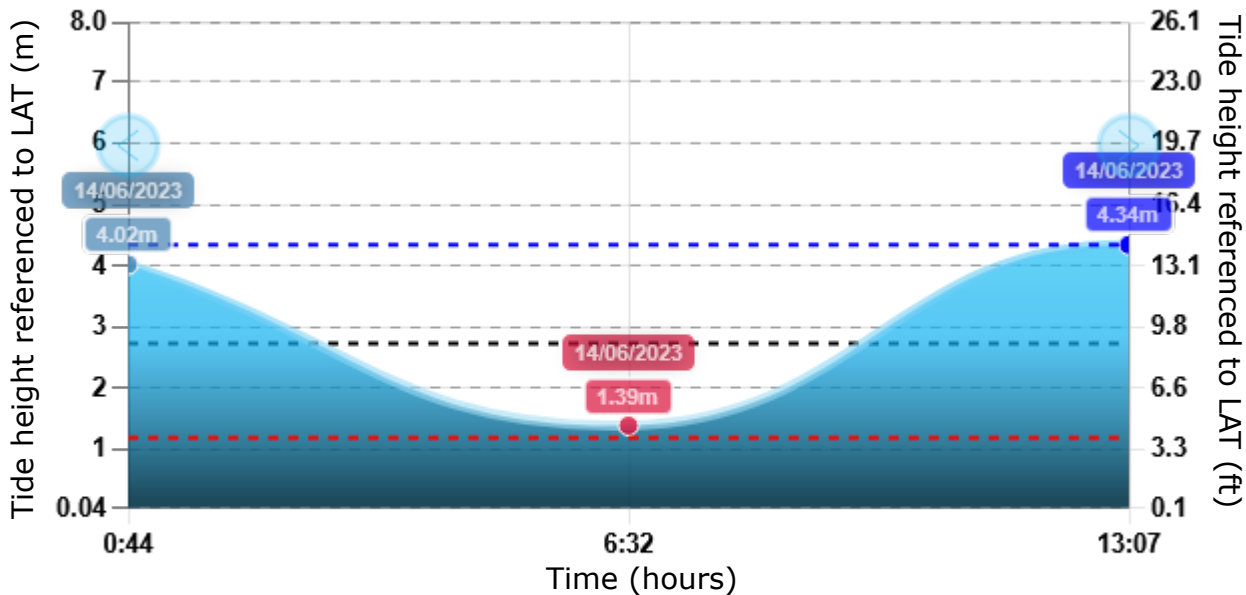

**Relevant information of the day**

**maximum height 4.34m**

**mean sea level 2.73m**

**minimun height 1.19m**

Supplement: Supplemental Information 6 — Data available at the Dimar (General Directorate of Maritime Ports of Colombia): https://www.dimar.mil.co/. [file peerj-13-19728-s006.pdf]

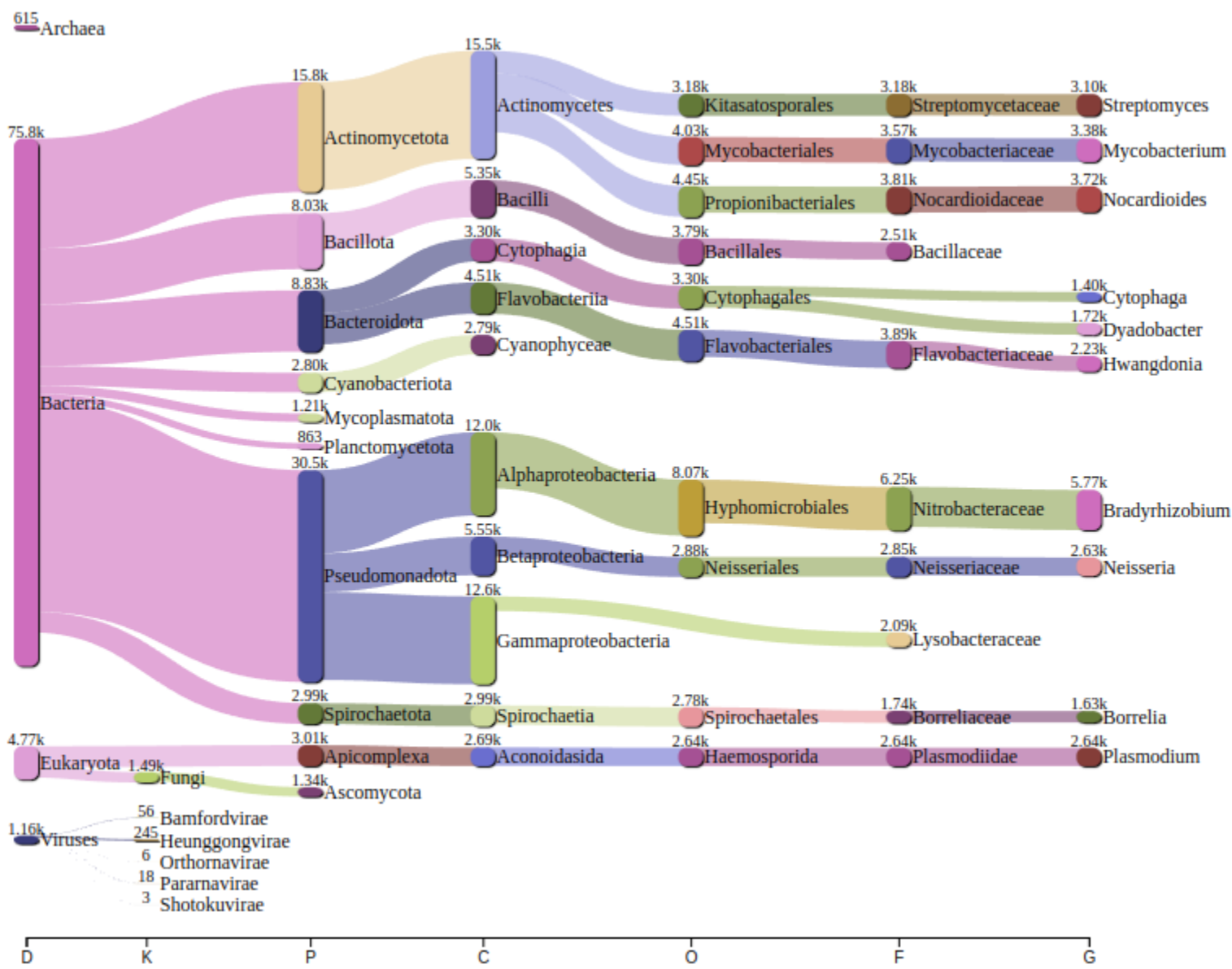

Supplement: Supplemental Information 8 [file peerj-13-19728-s008.pdf]

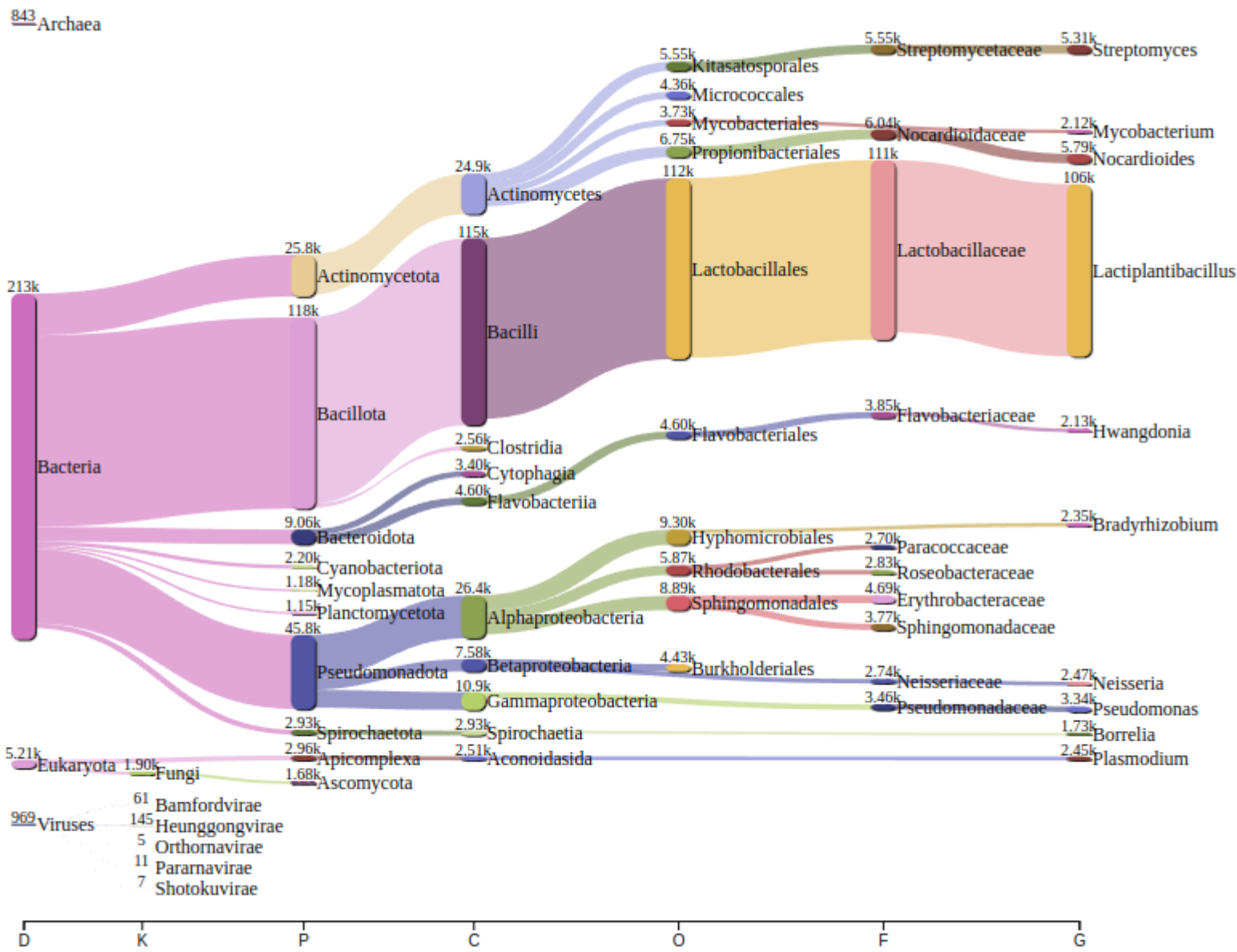

Supplement: Supplemental Information 9 [file peerj-13-19728-s009.pdf]

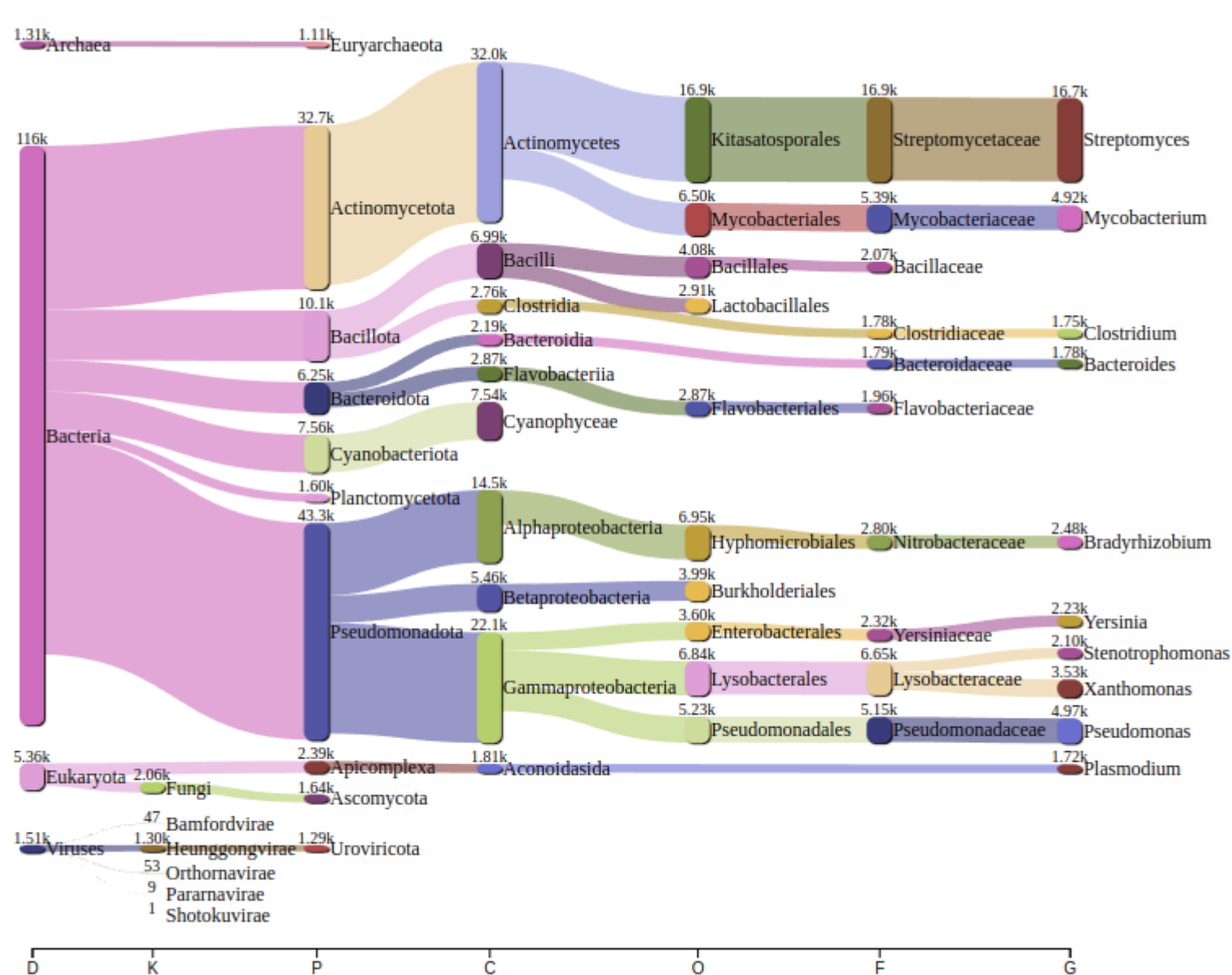

Supplement: Supplemental Information 10 [file peerj-13-19728-s010.pdf]

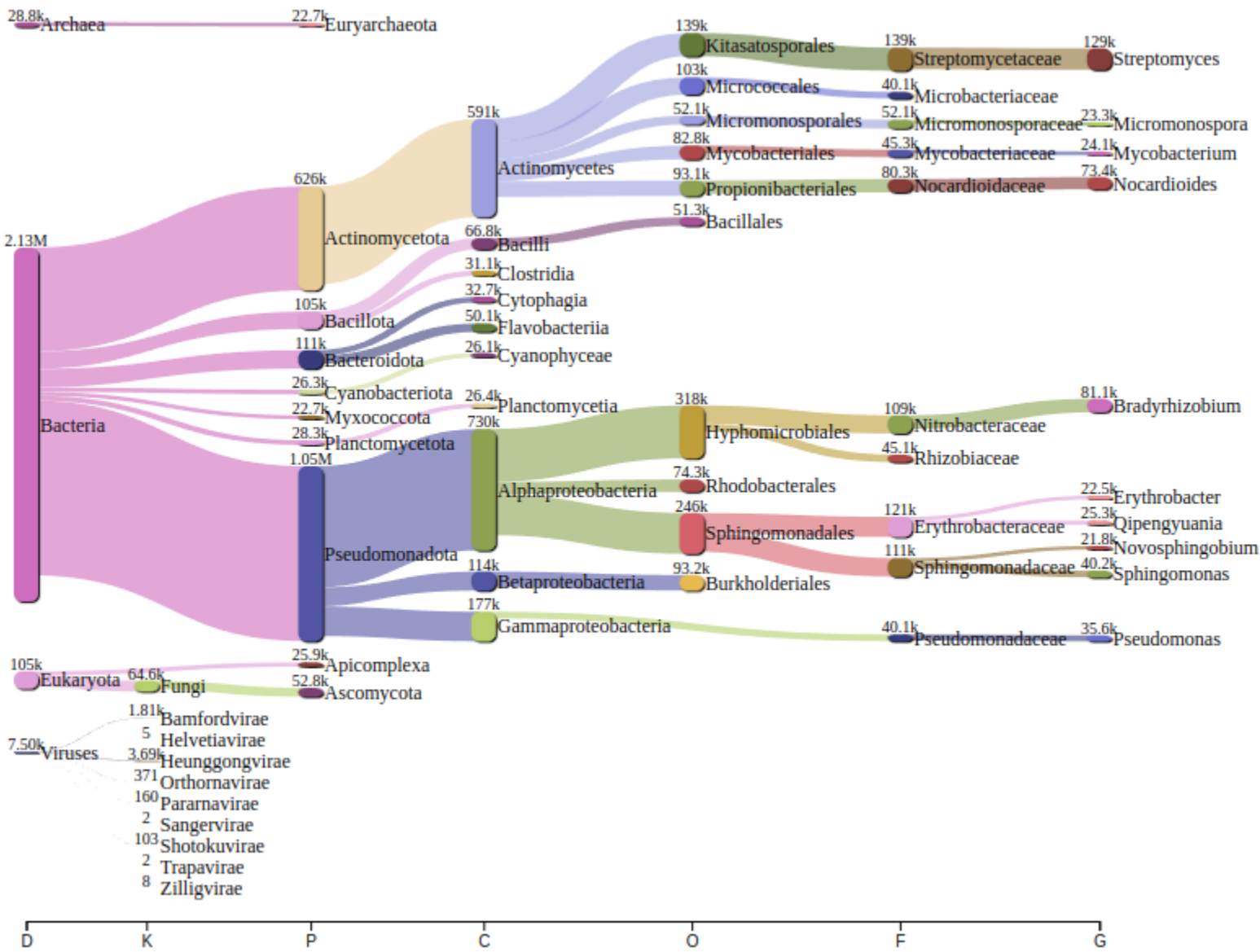

Supplement: Supplemental Information 11 [file peerj-13-19728-s011.pdf]

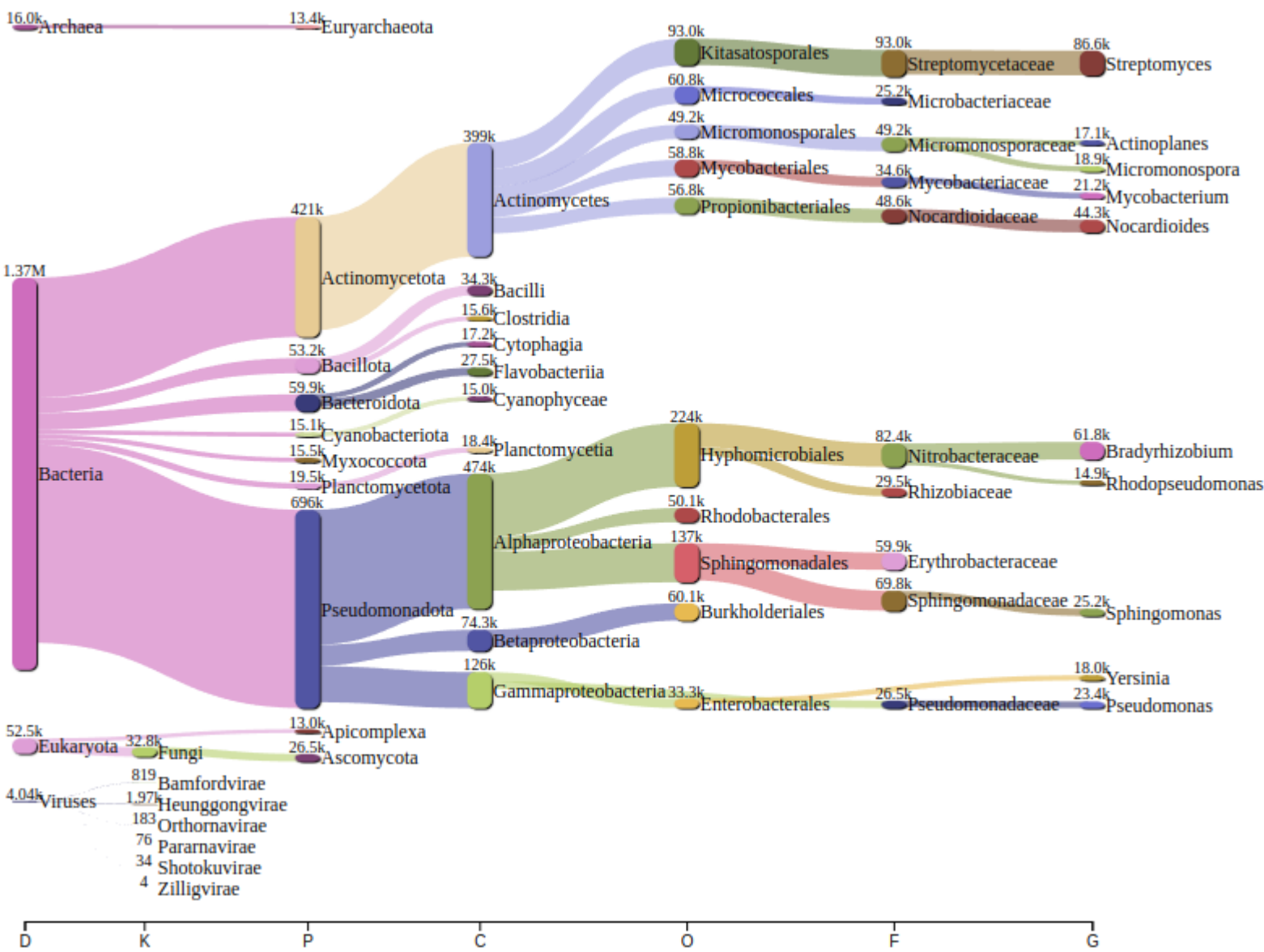

Supplement: Supplemental Information 12 [file peerj-13-19728-s012.pdf]

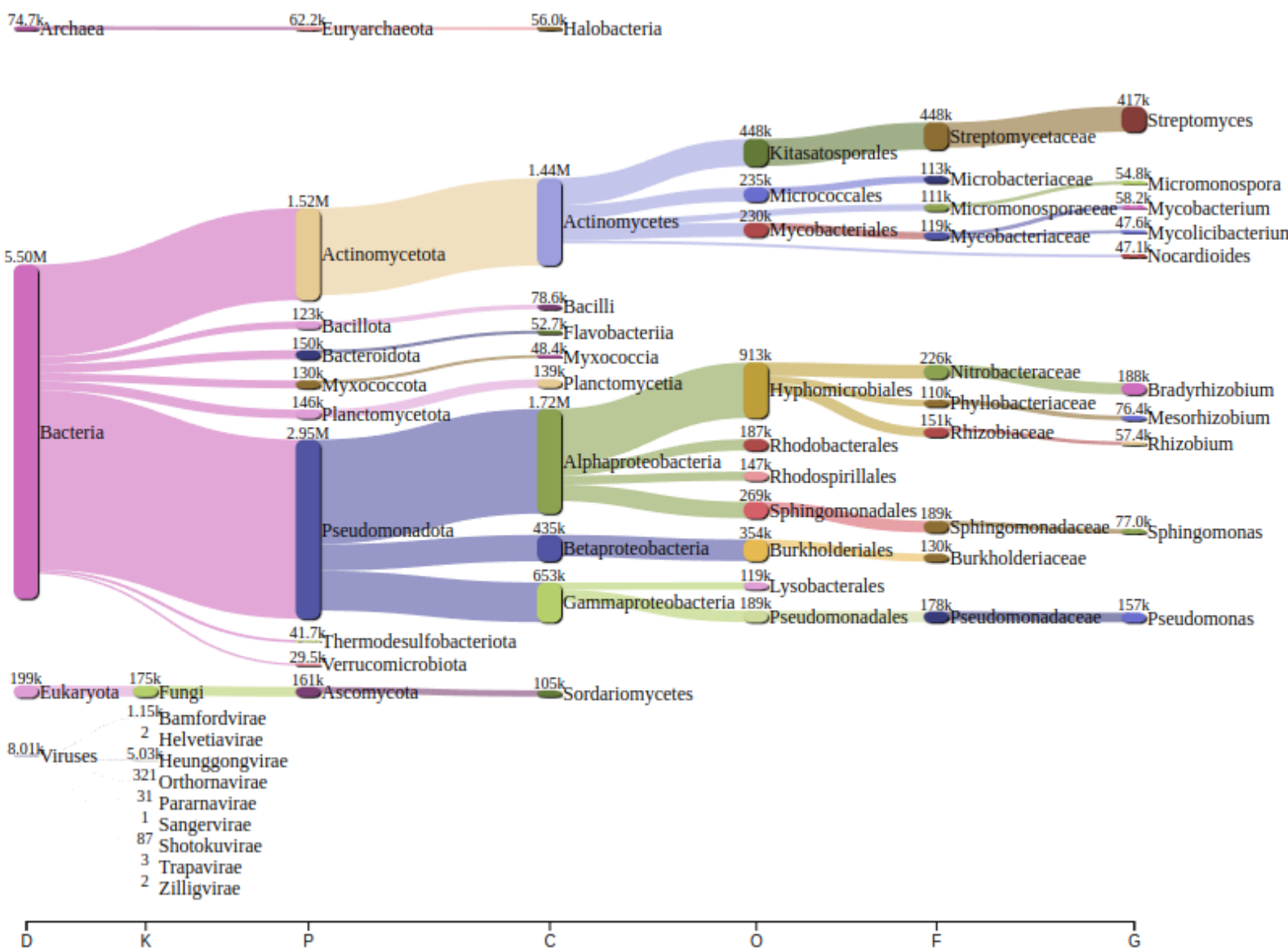

Supplement: Supplemental Information 13 [file peerj-13-19728-s013.pdf]

Alpha Diversity Measure

Observed

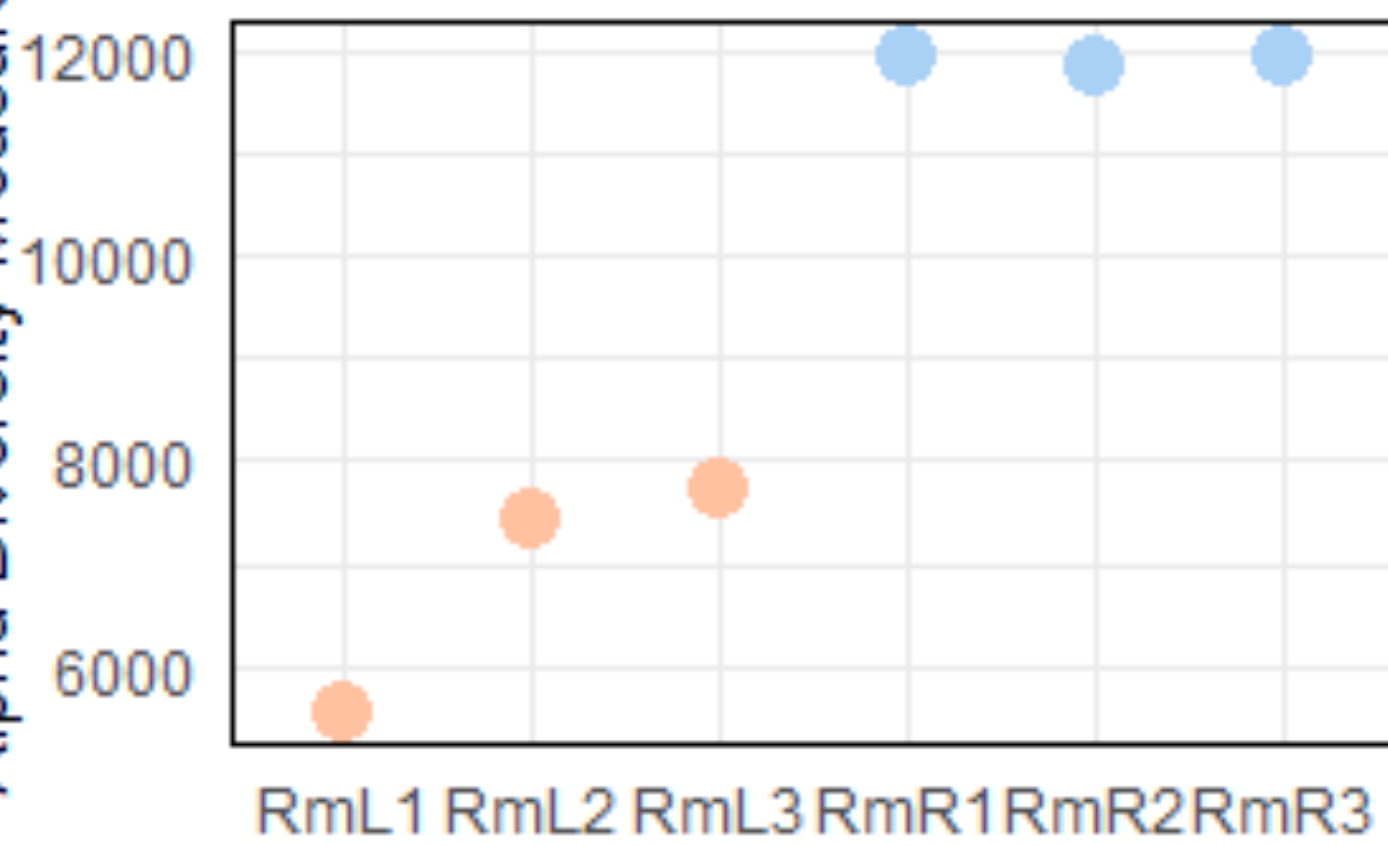

Shannon

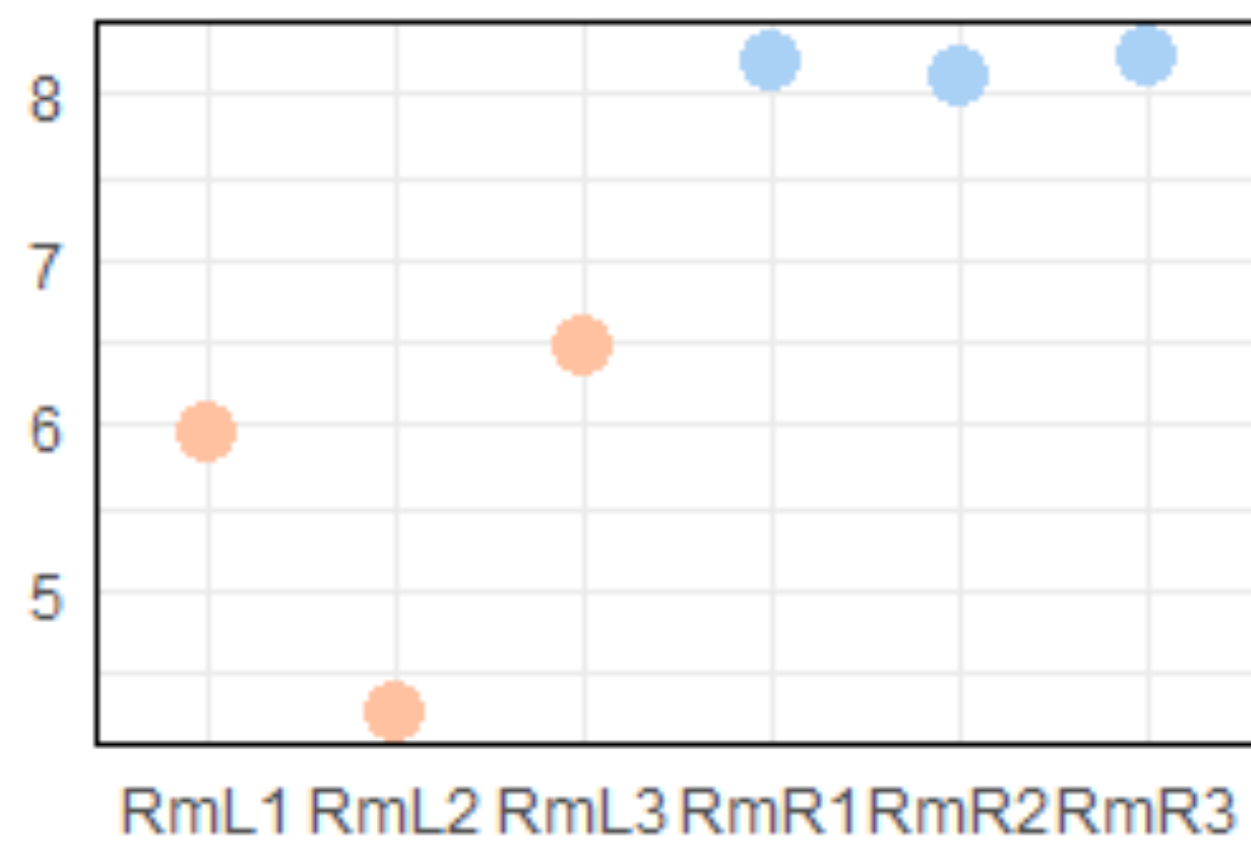

Simpson

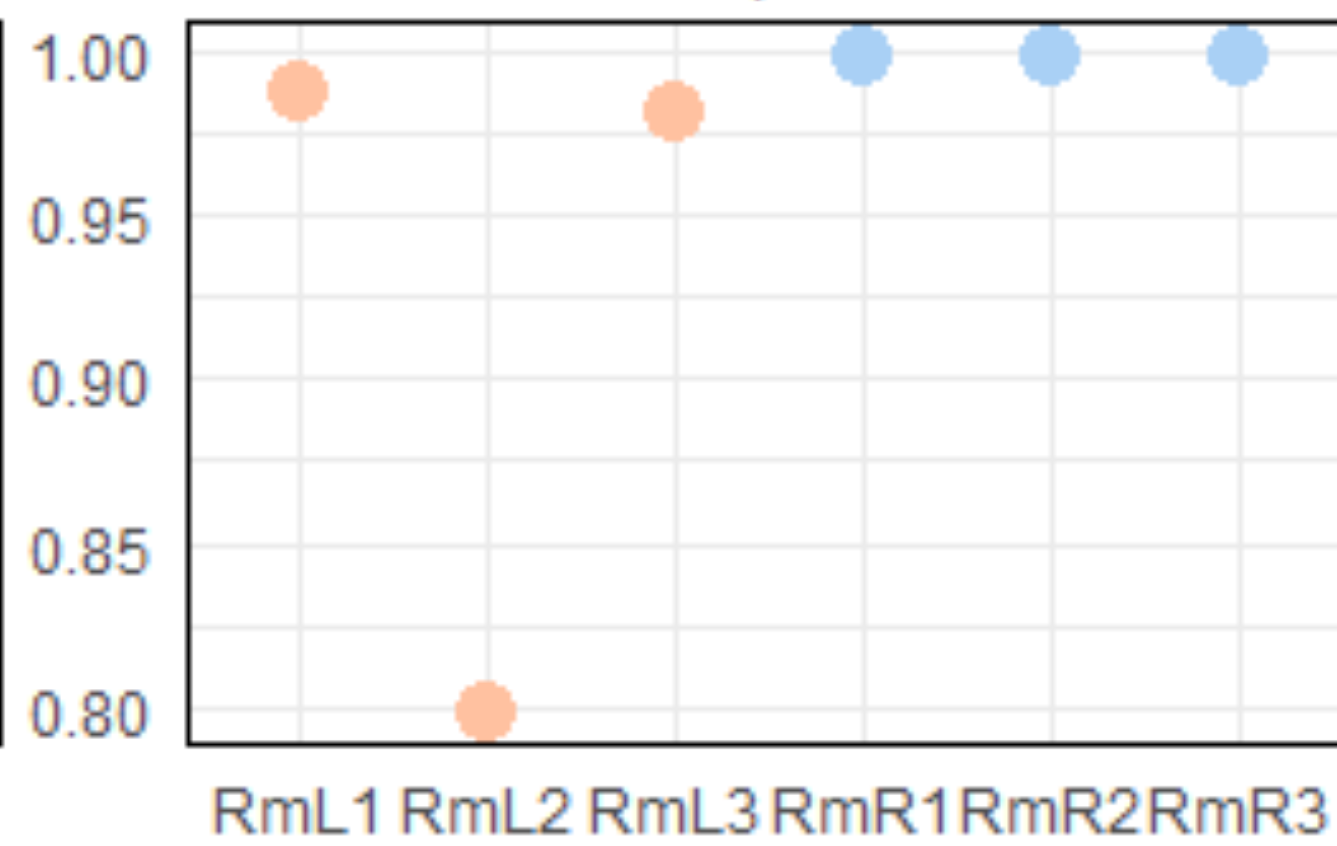

Tissue

Leaf  
Root

samples

Supplement: Supplemental Information 14 [file peerj-13-19728-s014.pdf]

A

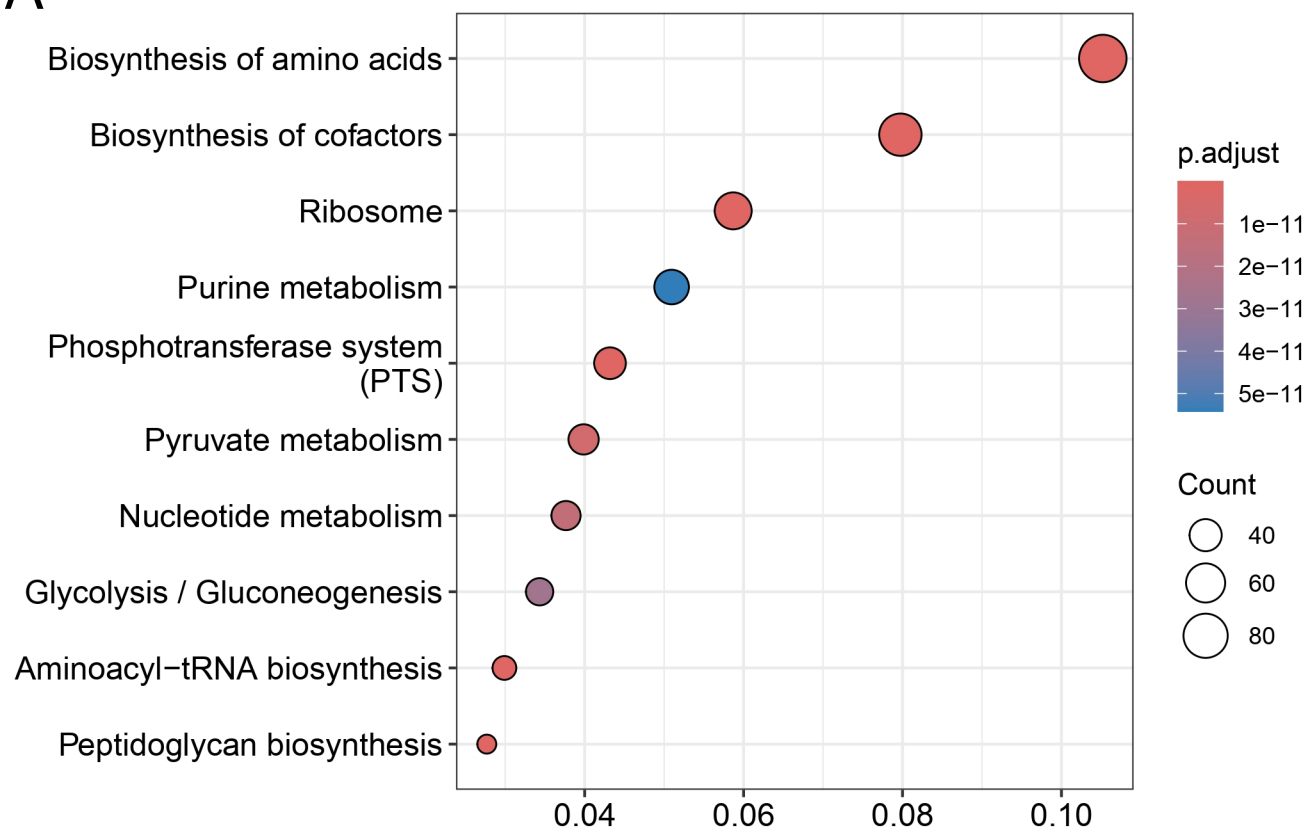

B

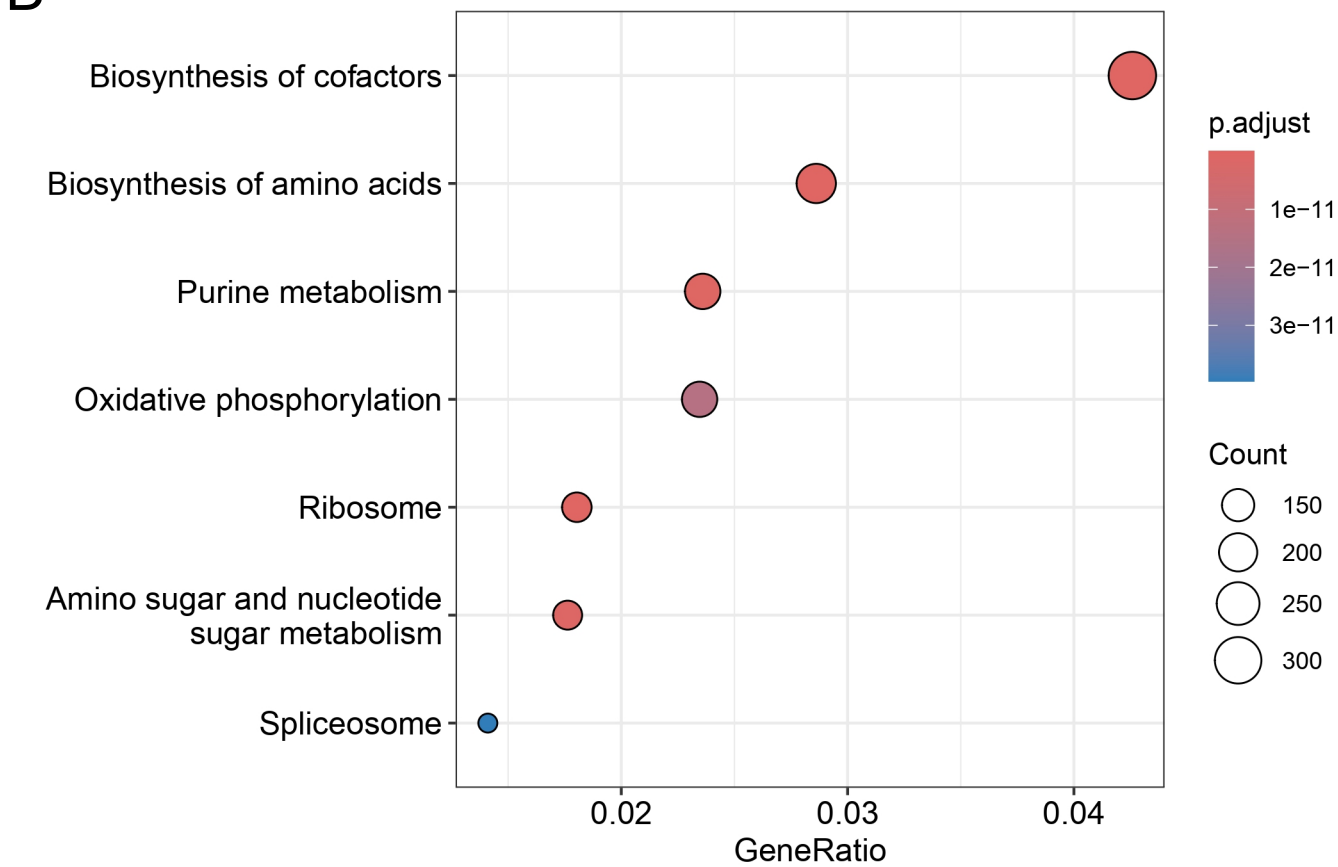

Supplement: Supplemental Information 15 — A) Metagenome of leaf tissue. B) Metagenome of root tissue. [file peerj-13-19728-s015.pdf]

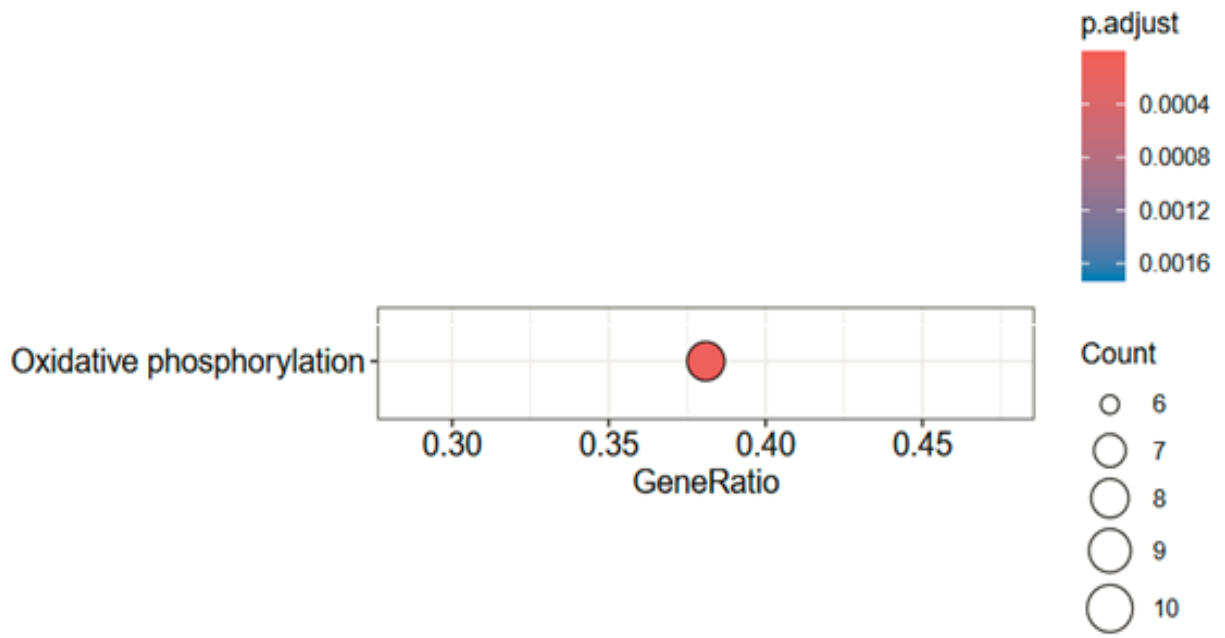

Supplement: Supplemental Information 16 [file peerj-13-19728-s016.pdf]
