# Supplementary material for: Uncovering tissue-specific endophytic microbiota composition and activity in Rhizophora mangle L.: a metagenomic and metatranscriptomic approach
Source: PeerJ. 2025 Aug 28;13:e19728. doi: 10.7717/peerj.19728 (PMC12399087; doi:10.7717/peerj.19728)
Supplement: Supplemental Information 7 — Three replicates per tissue type (leaf: RmL1–3; root: RmR1–3). DNA and RNA were extracted from each individual sample for metagenomic and meta-transcriptomic analyses. [file peerj-13-19728-s007.pdf]

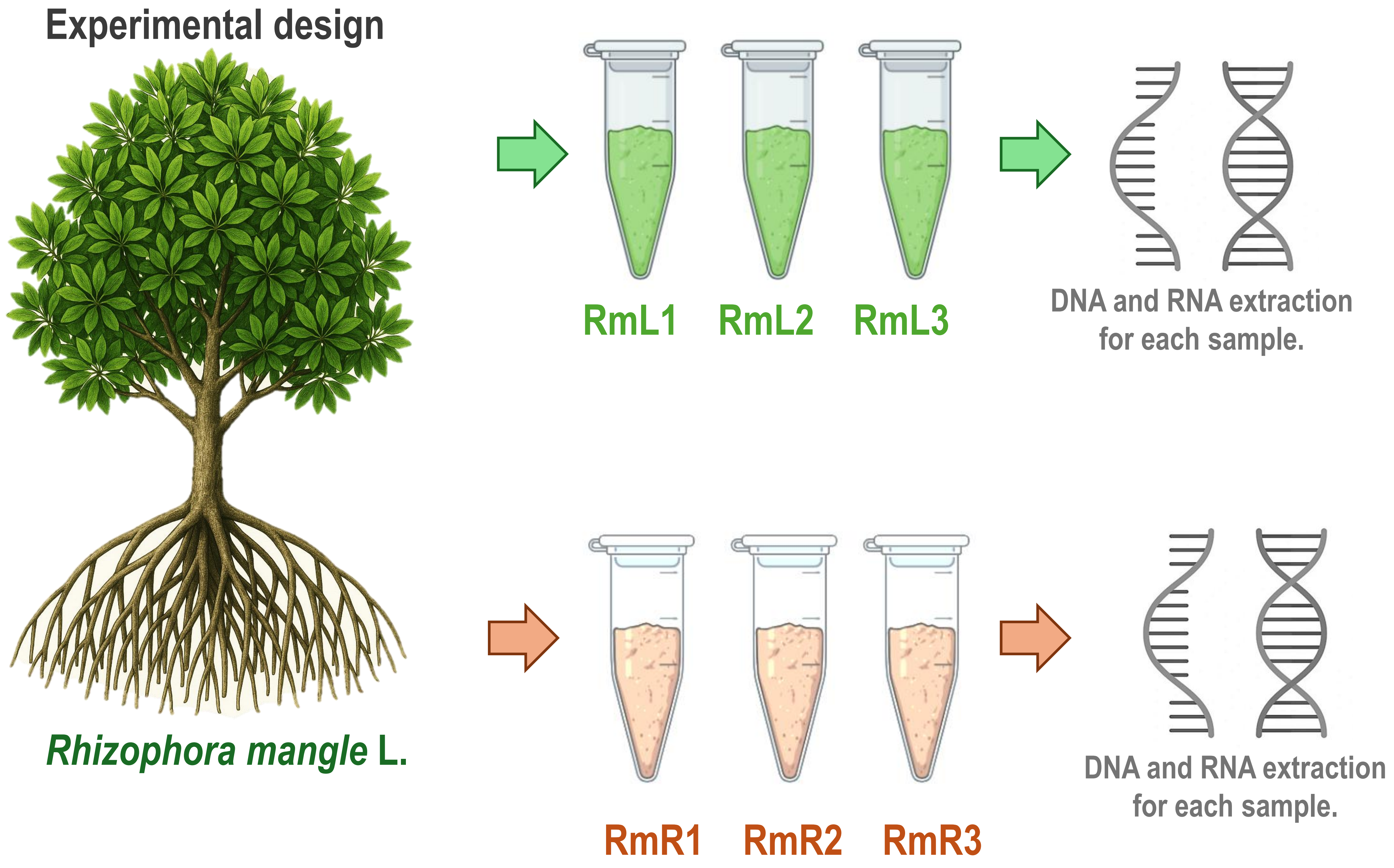
Experimental design
RmL1
RmL2
RmL3
DNA and RNA extraction for each sample.
Rhizophora mangle L.
RmR1
RmR2
RmR3
DNA and RNA extraction for each sample.

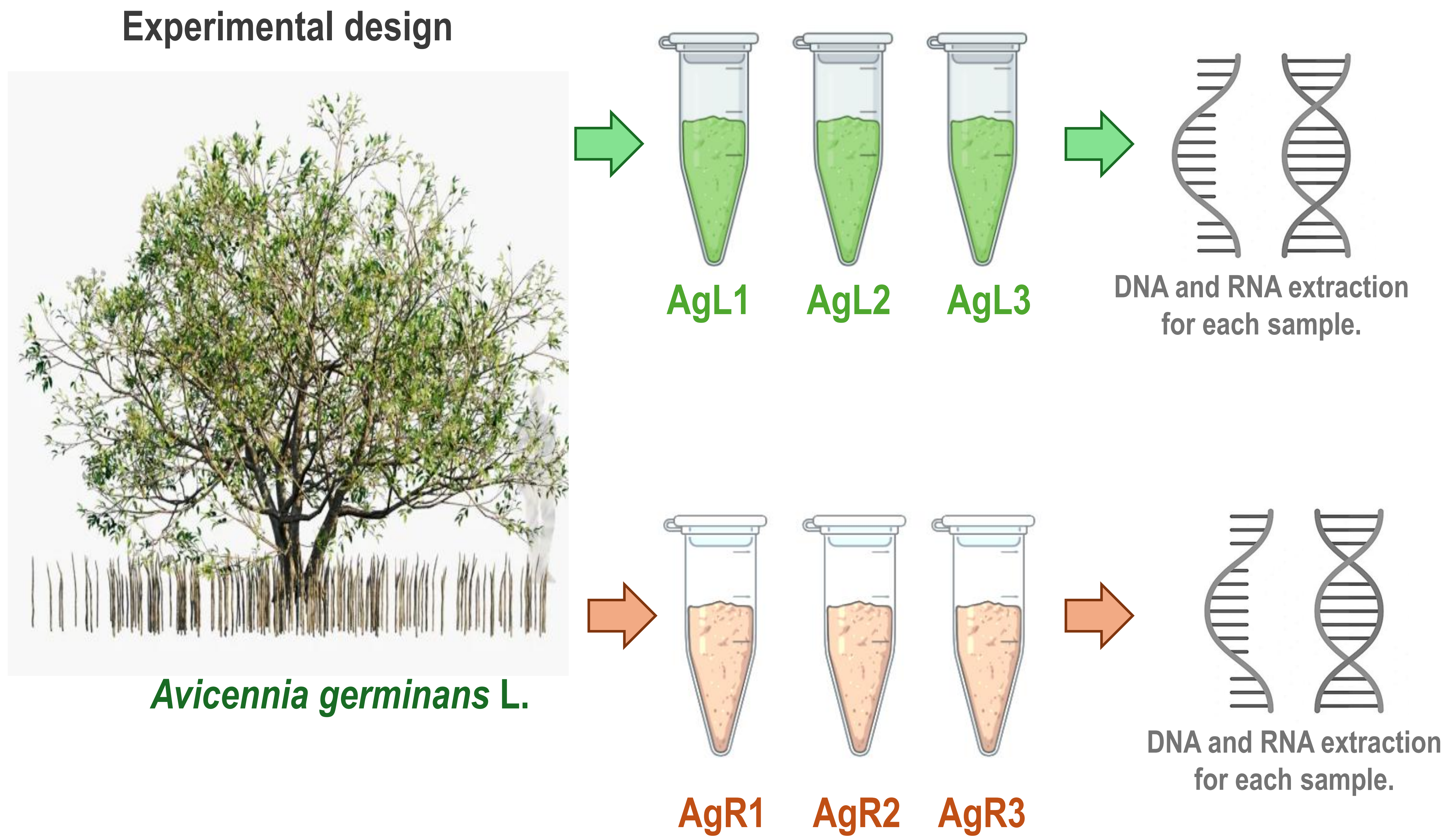
Experimental design
Avicennia germinans L.
AgL1
AgL2
AgL3
DNA and RNA extraction for each sample.
AgR1
AgR2
AgR3
DNA and RNA extraction for each sample.
